# Estimation of the hemoglobin glycation rate constant

**DOI:** 10.1038/s41598-020-80024-7

**Published:** 2021-01-13

**Authors:** Masashi Kameyama, Toshika Okumiya, Shinji Tokuhiro, Yoshihisa Matsumura, Hirotaka Matsui, Yasuhiro Ono, Tsuyoshi Iwasaka, Kazuyuki Hiratani, Masafumi Koga

**Affiliations:** 1 Department of Diagnostic Radiology, Tokyo Metropolitan Geriatric Hospital and Institute of Gerontology, Tokyo, 173-0015 Japan; 2 Department of Biomedical Laboratory Sciences, Faculty of Life Sciences, Kumamoto University, Kumamoto, 862-0976 Japan; 3 Department Clinical Laboratory, Kochi Medical School Hospital, Kochi, 783-8505 Japan; 4 Department of Laboratory Medicine, Kochi Medical School, Kochi, 783-8505 Japan; 5 Department of Molecular Laboratory Medicine, Faculty of Life Sciences, Kumamoto University, Kumamoto, 860-8556 Japan; 6 Department of Internal Medicine, Kouhoukai Takagi Hospital, Fukuoka, 831-0016 Japan; 7 Preventive Medical Center, Kouhoukai Takagi Hospital, Fukuoka, 831-0016 Japan; 8 Diabetes Center, Shinseikai Toyama Hospital, Toyama, 939-0243 Japan; 9 Department of Internal Medicine, Hakuhokai Central Hospital, Hyogo, 661-0953 Japan

**Keywords:** Diabetes, Diagnostic markers

## Abstract

In a previous study, a method of obtaining mean erythrocyte age ($M_{RBC}$) from HbA1c and average plasma glucose (AG) was proposed. However, the true value of the hemoglobin glycation constant ($k_g$ dL/mg/day), required for this model has yet to be well characterized. Another study also proposed a method of deriving $M_{RBC}$ from erythrocyte creatine (EC). Utilizing these formulae, this study aimed to determine a more accurate estimate of $k_g$. One hundred and seven subjects including 31 patients with hemolytic anemia and 76 subjects without anemia were included in this study. EC and HbA1c data were analyzed, and $M_{RBC}$ using HbA1c, AG and the newly-derived constant, $k_g$ were compared to $M_{RBC}$ using traditional $^{51}\hbox {Cr}$ in three patients whose data were taken from previous case studies. A value of $7.0\times 10^{-6}$ dL/mg/day was determined for $k_g$. $M_{RBC}$ using HbA1c, AG and $k_g$ were found to no be significantly different (paired *t*-test, $p=0.45$) to $M_{RBC}$ using traditional $^{51}\hbox {Cr}$. $k_g$ enables the estimation of $M_{RBC}$ from HbA1c and AG.

## Introduction

**Figure 1:**
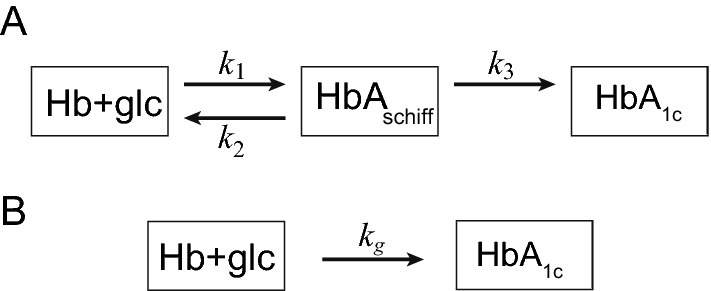
Hemoglobin glycation. (**A**) HbA1c is produced from Schiff base by amadori rearrangement. (**B**) Simplified two compartment model. $\hbox {HbA}_{\mathrm{schiff}}$ is aldimine complex (intermediate product). $k_1, k_2, k_3$, $k_g$ are kinetic constants.

HbA1c is widely used as both an indicator of glycemic control, as well as a diagnostic index, for diabetes in clinical settings^1,2^. Hemoglobin glycation is assumed to obey a three compartment model (Fig. 1). The rate constant of the total glycation reaction ($k_g$) is as follows.

1 $$\begin{aligned} k_g=\frac{k_1k_3}{k_{2} + k_3} \end{aligned}$$

Although HbA1c is generally indicative of recent glycemic control over the past 1–2 months, it is known to show reduced correlation to glycemic control status in the presence of diseases which result in a shortened erythrocyte lifespan such as hemolytic anemia^3^.

Erythrocyte creatine (EC) is a good marker that reflects the mean erythrocyte age^4^. We proposed a method that compensates glycated albumin (GA)/IFCC-HbA1c ratio for hemolysis by EC^5^.

We have recently proposed a simple method to obtain mean erythrocyte age ($M_{RBC}$) from HbA1c and average glucose (AG)^6^, which has theoretically derived based on $\Gamma$-like function model of erythrocyte lifespan^7^:

2 $$\begin{aligned} M_{RBC} \simeq \frac{HbA1c}{(1 - \frac{2}{3} HbA1c) k_g \mathrm{AG}} \end{aligned}$$

This formula provides meaningful information for the diagnosis of anemia. We estimated $k_g$ to be 6–10$\times 10^{-6}$ dL/mg/day based on past literature^6^. However, a more accurately estimated value of $k_g$ would provide more useful information.

The relationship between $M_{RBC}$ and EC was previously established based on a model and the data^8^ from 21 patients, which included EC and $^{51}\hbox {Cr}$, as following^9^:

3 $$\begin{aligned} M_{RBC} = -22.84 \log _e EC + 65.83 \end{aligned}$$

This study aimed to determine the accurate value of $k_g$ from EC-derived $M_{RBC}$ and HbA1c.

## Results

### Participant characteristics

Participant demographics are shown in Table 1. All participants had no more than 16% GA. There was no significant difference in the GA of anemic and non-anemic subjects. However HbA1c, Hb, EC and their derivatives showed significant variation between the two groups.

The demographic information on the 3 patients from the previous cases are shown in Table 2.

**Table 1:** Participants characteristics.

	Non-hemolysis	Hemolysis	*p*
*n* (M/F)	76 (30/46)	31 (17/14)	0.1463
Age (years)	62.3 ± 7.9	45.6 ± 15.0	$1.37\times 10^{-6}$
HbA1c (%)	$5.78 \pm 0.25$	$4.05\pm 0.78$	$2.27\times 10^{-13}$
iA1c (mmol/mol)	$39.7\pm 2.7$	$20.8 \pm 8.5$	$2.27\times 10^{-13}$
GA (%)	$13.57 \pm 1.07$	$13.06 \pm 1.75$	0.151
GA/iA1c	$0.343 \pm 0.032$	$0.762 \pm 0.363$	$5.85\times 10^{-7}$
Hb (g/dL)	$14.26 \pm 1.16$	$9.75 \pm 1.97$	$2.44\times 10^{-14}$
EC (μmol/g Hb)	$1.40 \pm 0.21$	$5.47 \pm 2.13$	$1.42\times 10^{-11}$
EC-$$M_{RBC} $$ (days)	$$58.5 \pm 3.41$$	$$29.0 \pm 10.0$$	$$4.46\times 10^{-17}$$

Results are expressed as mean ± standard deviation (SD). Sex ratio was examined by $\chi ^2$ test. Other items were examined by *t*-test (bilateral).

GA, glycated albumin; EC, erythrocyte creatine.

**Table 2:** Characteristics of three reported patients with latent hemolysis and DM in literature.

Case	Herranz^10^	Ishii^11^	Hiratani^12^
Age/sex	30F	72M	58F
Disease	AIHA	AIHA	HSt
DM	Type 1	Type 2	Type 2
HbA1c (%)	5.4	6.5	5.8
GA (%)	–	26.1	23.3
Hb (g/dL)	Normal	13.5	11.5
Ret (%)	Normal	1.3	1.3
Hpt (mg/dL)	Normal	82	58

These patients showed normal Hb, reticulocyte, and haptogloblin.

AIHA, autoimmune hemolytic anemia; HSt, hereditary stomatocytosis, DM, diabetes mellitus; GA, glycated albumin; Hb, hemoglobin; Ret, reticulocyte; Hpt, haptoglobin.

### Estimation of $k_g$

**Figure 2:**
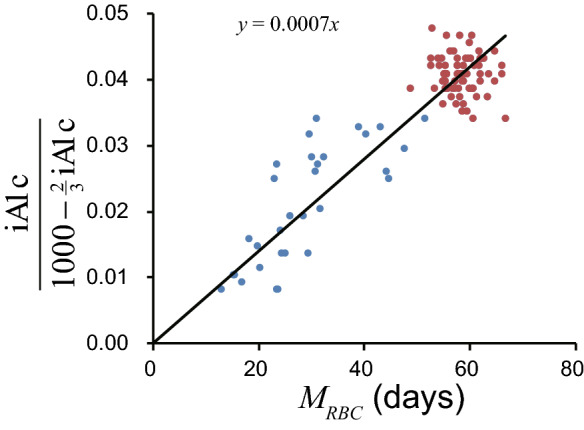
Relationship between EC derived $M_{RBC}$ and iA1c/(1000−(2/3)iA1c). Red circles denote non-hemolytic participants and blue circles denote hemolytic patients. Black line denotes regression line through origin.

EC derived $M_{RBC}$ and $\frac{\mathrm{iA1c}}{1000- \frac{2}{3}{\mathrm{iA1c}}}$ are shown in Fig. 2. A linear relationship was successfully observed.

$k_g$ calculated by the two methods outlined previously, for non-hemolytic participants and the entire study population are seen in Table 3. All 4 numbers can be approximated to $7\times 10^{-6}$. Figure 2 shows that data from severe hemolytic patients is less stable. Thus, the value derived from the direct method for calculating $k_g$ is likely to be the least accurate. Excluding this value as an outlier, the 3 remaining figures were 6.94–6.99 $\times 10^{-6}$ (average $6.970\times 10^{-6}$). Therefore, considering significant figures, $k_g$ can be said to be $7.0\times 10^{-6}$.

**Table 3:** $$k_g$$ estimation.

Population	Slope	Straight forward
The whole	$6.973\times 10^{-6}$	($7.073 \pm 1.229) \times 10^{-6}$
Non-hemolytic	$6.942\times 10^{-6}$	($6.994 \pm 0.662) \times 10^{-6}$

Results obtained by the direct method are expressed as mean ± standard deviation (SD).

### Confirmation of derived $k_g$

The $M_{RBC}$ using the derived $k_g$, $7.0\times 10^{-6}$ and $M_{RBC}$ using $^{51}\hbox {Cr}$ half-life are shown in Table 4.

$M_{RBC}$ derived from iA1c were $36.95\pm 5.93$, $M_{RBC}$ derived from $^{51}\hbox {Cr}$ half-life were $41.29\pm 2.22$. Paired *t*-test: *t*, $-0.9278$; df, 2; *p* (bilateral), 0.4514. Thus, $M_{RBC}$ derived from iA1c and $M_{RBC}$ using $^{51}\hbox {Cr}$ half-life were not significantly different.

**Table 4:** $M_{RBC}$ of 3 cases in literature.

Case	Herranz^10^		Ishii^11^		Hiratani^12^
HbA1c (%)	5.8	4.9	6.0	6.7	6.5
iA1c (mmol/mol)	39.9	30.0	42.3	50.2	47.5
AG (mg/dL)	203	148	138	177	184
iA1c derived (days) $M_{RBC}$	28.7	29.6	45.3	41.9	38.1
	29.2		43.6		38.1
$^{51}\hbox {Cr}$ half-life (days)	20		20		17.8
$^{51}\hbox {Cr}$ derived $M_{RBC}$ (days)	42.9		42.9		38.1

HbA1c value is different from Table 2. The AG in Herranz^10^ and Ishii^11^ were calculated from blood glucose values using self-monitoring of blood glucose (SMBG).

## Discussion

Based on EC-derived $M_{RBC}$ and HbA1c data, a more accurate value for the constant $k_g$ was obtained. Though $k_g$ was previously determined to be 6–10 $\times 10^{-6}$ dL/mg/day^6^, the more accurate value of $7.0\times 10^{-6}$ improves the usefulness of the proposed model allowing closer approximation of $M_{RBC}$ based on AG and iA1c.

Moreover, the validity of $k_g$ has been confirmed through comparison of $M_{RBC}$ derived from iA1c and $k_g$ with $M_{RBC}$ derived from $^{51}\hbox {Cr}$ half-life. Of the three patients with hemolytic anemia and comorbid DM analyzed, data from two patients showed a remarkable correlation with the model derived figures. Data from one patient showed a 1.47 times difference in values however, this may be attributable to the use of SMBG instead of CGM, and the difficulty of standardizing $^{51}\hbox {Cr}$ data containing elution.

Variant hemoglobin should be distinguished from hemolysis when $M_{RBC}$ determined by Eq. (2) is low. Glycated variant hemoglobin will exhibit different peaks in HPLC from normal HbA1c, resulting in erroneously low values for HbA1c (some variants show an artefactually high value). It has previously been reported that variant hemoglobin can be detected by the dissociation between HbA1c measured by HPLC and by immunoassay^13^. Moreover, some variant hemoglobins such as Hb Himeji^14^ have different $k_g$ values from normal Hb. In patients with these variant hemoglobins, Eq. (2) is likely to provide a falsely low $M_{RBC}$.

There are a number of limitations to this study. The data used to calculate a more specific estimate of $k_g$ contained EC and HbA1c, but lacked CGM data, necessitating the use of 100 mg/dL as an approximation of AG. However, participants were confirmed to be free of DM through GA, an indicator of glycemic control that is independent of mean erythrocyte age, with a cut off of GA no more than 16%. Further study with more complete data including CGM, HbA1c and EC would provide an even more definitive value for $k_g$. Another limitation is that the value for $k_g$ derived in this study is totally dependent on Eq. (3) that derives $M_{RBC}$ from EC. This equation was based on old published data^8^, which used less sensitive and poorly specific chemical methods of measuring creatine which were prone to cross-reactivity with other guanidino compounds. This may reduce the reliability of the system. In contrast, in this study creatine was measured using an enzymatic method which was sensitive and specific to creatine in erythrocytes which uses 10-*N*-methylcarbamoyl-3,7-*bis*(dimethylamino) phenothiazine (MCDP), an *N*-methylcarbamoyl derivative of methylene blue, with a high molar absorption coefficient ($9.6\times 10^7\hbox {L mol}^{-1}\,\hbox {cm}^{-1}$)^4^, as a chromogen.

## Methods

### Participants

One hundred and seven subjects including 31 patients with hemolytic anemia and 76 subjects without anemia were included in this study. All samples were prepared and analyzed in accordance with the protocols approved by the institutional committees at Kumamoto University and other collaborating institutions.

Patients with hemolytic anemia were recruited from 115 patients who were older than 20 years old and required laboratory tests including complete blood counts and reticulocyte counts (Ret) for clinical reasons. Those who were suspected of having diabetes mellitus (DM) based on history, a low 1,5-Anhydroglucitol (1,5-AG) value (male, < 14.9 μg/mL; female, < 12.4 μg/mL), or had comorbid liver or renal diseases, were excluded, as liver and renal diseases affect HbA1c and GA. EC, HbA1c, GA, haptoglobin, and other biochemical screening items were measured using the existing plasma samples from these patients. Use of existing plasma samples from anemic patients without written consent was approved by the institutional review board.

Participants without anemia were recruited from medical examination checkup recipients at Takagi Hospital. Those who had anemia, DM, liver disease, renal disease or who were pregnant were excluded to avoid confounding effects on HbA1c or GA value. We provided the healthy volunteers with detailed information about the study, and all participants without anemia provided written informed consent to participate.

### Data interpretation

EC was measured enzymatically in accordance with a previous report^4^, HbA1c was measured by high performance liquid chromatography (HPLC) method^15^, and GA was measured by enzymatic method using albumin-specific protenase, ketoamine oxidase, and albumin assay reagent (Lucica GA-L; Asahi Kasei Pharma Co., Tokyo, Japan)^16^.

HbA1c expressed in International Federation of Clinical Chemistry (IFCC) units (iA1c) was used for calculations in this study. While the National Glycohemoglobin Standardization Program (NGSP) is used to express HbA1c in many clinical research and medical care settings, NGSP is measured by an old standardized method and at the time of conception, HPLC was not able to distinguish true HbA1c from other products. HPLC technology later advanced, however the derived HbA1c value is adjusted to NGSP in the interest of consistency. IFCC provides a strict definition of iA1c as hemoglobin with a glycated valine in the N-terminal $\beta$-chain. Thus, iA1c value is preferred value for estimation of hemoglobin glycation.

To acquire iA1c from HbA1c expressed in NSGP unit, we used the following equation^17^:

4 $$\begin{aligned} \mathrm{HbA1c}_{\mathrm{NGSP}} \, (\%)= & {} 0.0915 \times \mathrm{iA1c} \, (\mathrm{mmol/mol}) + 2.153 (\%) \end{aligned}$$

5 $$\begin{aligned} \iff \mathrm{iA1c} (\mathrm{mmol/mol})= & {} 10.93 \times \mathrm{HbA1c}_{\mathrm{NGSP}} \, (\% ) - 23.53 \end{aligned}$$

$M_{RBC}$ was acquired from EC by the aforementioned Eq. (3).

An AG value of 100 mg/dL was substituted for plasma glucose values derived using CGM. This number was based on the average AG of non-diabetic participants and the previously reported findings from a study which showed the median AG in healthy subjects to be reported to be 101.0 (96.3–106.0) mg/dL^18^ and another ADAG (A1c-derived average glucose) study which found that the AG of the non-diabetic group of their study was similarly 100 mg/dL^19,20^.

$M_{RBC}$ was also determined using $^{51}\hbox {Cr}$ half-life. As the reference range for $^{51}\hbox {Cr}$ half-life was described as 28–30 days^10^, 30 ± 5 days^11^, and 26–40 days^12^, $M_{RBC}$ was calculated by multiplying $^{51}\hbox {Cr}$ half-life and 2.14 (= 60/28), 60 days being the normal value for $M_{RBC}$.

### Data analysis

*EC* and $$M_{RBC} $$ data were analyzed using a spreadsheet software, Excel 365 (Microsoft Corporation, Redmond, WA, USA).

### Estimation of $k_g$

The following two methods were used to estimate $k_g$. The slope method—the following Eq. (6) derived from Eq. (2) shows that the slope of the line connecting a point and the origin is $k_g \mathrm{AG}$.

6 $$\begin{aligned} \frac{\mathrm{iA1c}}{1000 - \frac{2}{3} {\mathrm{iA1c}} } = k_g \mathrm{AG} \times M_{RBC} \end{aligned}$$

Estimating the slope of the regression line through the origin by the least square model:

7 $$\begin{aligned} \frac{\sum _i^n x_iy_i }{\sum _i^n x_i^2 } \end{aligned}$$

where $x_i$, $y_i$ are $M_{RBC}$ and $\frac{\mathrm{iA1c}}{1000- \frac{2}{3}{\mathrm{iA1c}}}$ of each participant, respectively.

The direct method—the $k_g$ of each participant was calculated by the following equation:

8 $$\begin{aligned} k_g = \frac{\mathrm{iA1c}}{(1000 - \frac{2}{3} {\mathrm{iA1c}}) M_{RBC} {\mathrm{AG} }} \end{aligned}$$

Then, average and standard deviation of each $k_g$ was calculated.

### Confirmation of derived $k_g$

The method of obtaining $M_{RBC}$ from AG and iA1c was applied to data from three patients with latent hemolysis who were presented in a previous case studies^10–12^.

Data of Herranz^10^ and Ishii^11^ showed changes in HbA1c during the course of the study. Therefore, $M_{RBC}$ was calculated separately for each period. For the Ishii case^11^, AG was calculated by averaging self-monitoring of blood glucose (SMBG) data for each period. The Hiratani study^12^ examined $^{51}\hbox {Cr}$ erythrocyte lifespan measurement during hospitalization in Oct 1999 and CGM in Feb 2016. While HbA1c and plasma glucose concentrations fluctuate routinely, RBC lifespan remain comparatively constant, especially when influenced by a certain diseases (stomatocytosis). Furthermore, supply of $^{51}\hbox {Cr}$ was ceased in Japan in 2015 and thus it can no longer be used to study erythrocyte lifespan.

### Ethical approval and consent to participate

The work was conducted in accordance with Ethical Guidelines for Medical and Health Research Involving Human Subjects in Japan and conformed to the Helsinki Declaration. All samples were prepared and analyzed in accordance with the protocols approved by the institutional committees at Kumamoto University and other collaborating institutions.

## Data Availability

The data supporting the findings can be obtained on reasonable request to the corresponding author.

## Abbreviations

AG: Average plasma glucose
CGM: Continuous glucose monitoring
DM: Diabetes mellitus
EC: Erythrocyte creatine
GA: Glycated albumin
Hb: Hemoglobin
HPLC: High performance liquid chromatography
Hpt: Haptoglobin
IFCC: International Federation of Clinical Chemistry
$k_g$: Hemoglobin glycation constant
MCDP: 10-*N*-methylcarbamoyl-3,7-*bis* (dimethylamino) phenothiazine
$M_{RBC}$: Mean erythrocyte age
NGSP: National Glycohemoglobin Standardization Program
Ret: Reticulocyte
SMBG: Self-monitoring of blood glucose.
